# The state of health in the European Union (EU-27) in 2019: a systematic analysis for the Global Burden of Disease study 2019

**DOI:** 10.1186/s12889-024-18529-3

**Published:** 2024-05-22

**Authors:** João Vasco Santos, Alicia Padron-Monedero, Boris Bikbov, Diana Alecsandra Grad, Dietrich Plass, Enkeleint A. Mechili, Federica Gazzelloni, Florian Fischer, Gerhard Sulo, Che Henry Ngwa, Isabel Noguer-Zambrano, José L. Peñalvo, Juanita A. Haagsma, Katarzyna Kissimova-Skarbek, Lorenzo Monasta, Nermin Ghith, Rodrigo Sarmiento-Suarez, Rok Hrzic, Romana Haneef, Rónán O’Caoimh, Sarah Cuschieri, Stefania Mondello, Zubair Kabir, GBD 2019 EU State of Health Collaborators, Cristiana Abbafati, Hassan Abolhassani, Victor Adekanmbi, Keivan Ahmadi, Sepideh Ahmadi, Adel Al-Jumaily, François Alla, Jordi Alonso, Robert Ancuceanu, Catalina Liliana Andrei, Tudorel Andrei, Sofia Androudi, Josep M. Antó, Seth Christopher Yaw Appiah, Olatunde Aremu, Benedetta Armocida, Johan Ärnlöv, Ashokan Arumugam, Sameh Attia, Avinash Aujayeb, Marcel Ausloos, Jose L. Ayuso-Mateos, Maciej Banach, Till Winfried Bärnighausen, Francesco Barone-Adesi, Sandra Barteit, Sanjay Basu, Bernhard T. Baune, Massimiliano Beghi, Luis Belo, Derrick A. Bennett, Antonio Biondi, Mahdi Bohluli, Israel Júnior Borges do Nascimento, Nicola Luigi Bragazzi, Tasanee Braithwaite, Hermann Brenner, Danilo Buonsenso, Reinhard Busse, Daniela Calina, Giulia Carreras, Márcia Carvalho, Giulio Castelpietra, Alberico L. Catapano, Maria Sofia Cattaruzza, Joht Singh Chandan, Periklis Charalampous, Vijay Kumar Chattu, Simiao Chen, Rajiv Chowdhury, Hanne Christensen, Sheng-Chia Chung, Joao Conde, Barbara Corso, Natália Cruz-Martins, Giovanni Damiani, Alejandro de la Torre-Luque, Andreas K. Demetriades, Nikolaos Dervenis, Mostafa Dianatinasab, Diana Dias da Silva, Abdel Douiri, David Edvardsson, Luchuo Engelbert Bain, Francesco Esposito, Adeniyi Francis Fagbamigbe, Carla Sofia eSá Farinha, Seyed-Mohammad Fereshtehnejad, João C. Fernandes, Pietro Ferrara, Peter Andras Gaal, Silvano Gallus, Lucia Galluzzo, Mariana Gaspar Fonseca, Gus Gazzard, Alessandro Gialluisi, Simona Giampaoli, Paramjit Singh Gill, James C. Glasbey, Giuseppe Gorini, Michal Grivna, Abdul Hafiz, Josep Maria Haro, Jan Hartvigsen, Simon I. Hay, Behzad Heibati, David Hillus, Mehdi Hosseinzadeh, Mihaela Hostiuc, Sorin Hostiuc, Salman Hussain, Gaetano Isola, Olatunji Johnson, Jost B. Jonas, Tamas Joo, Jacek Jerzy Jozwiak, Mikk Jürisson, Marina Karanikolos, Joonas H. Kauppila, Moien A. B. Khan, Khaled Khatab, Miloslav Klugar, Ai Koyanagi, Om P. Kurmi, Dian Kusuma, Carlo La Vecchia, Ben Lacey, Demetris Lamnisos, Heidi Jane Larson, Anders O. Larsson, Savita Lasrado, Paolo Lauriola, Jeffrey V. Lazarus, Caterina Ledda, Paul H. Lee, Mall Leinsalu, Matilde Leonardi, Miriam Levi, An Li, Christine Linehan, Giancarlo Logroscino, Stefan Lorkowski, Joana A. Loureiro, Ronan A. Lyons, Áurea M. Madureira-Carvalho, Azeem Majeed, Alexander G. Mathioudakis, Colm McAlinden, John J. McGrath, Ritesh G. Menezes, Alexios-Fotios A. Mentis, Atte Meretoja, Tuomo J. Meretoja, Tomislav Mestrovic, Junmei Miao Jonasson, Bartosz Miazgowski, Tomasz Miazgowski, Andreea Mirica, Shafiu Mohammed, Ali H. Mokdad, Ute Mons, Joana Morgado-da-Costa, Francesk Mulita, Christopher J. L. Murray, Ionut Negoi, Ruxandra Irina Negoi, Serban Mircea Negru, Evangelia Nena, Nurulamin M. Noor, George Ntaios, Bogdan Oancea, Frank B. Osei, Adrian Otoiu, Raffaele Palladino, Songhomitra Panda-Jonas, Shahina Pardhan, Jay Patel, Mihaela Paun, Paolo Pedersini, Umberto Pensato, Renato B. Pereira, Jorge Pérez-Gómez, Norberto Perico, Ionela-Roxana Petcu, Carrie B. Peterson, Marina Pinheiro, Maarten J. Postma, Alberto Raggi, Amir Masoud Rahmani, Chythra R. Rao, Salman Rawaf, Reza Rawassizadeh, Giuseppe Remuzzi, Abanoub Riad, Simona Sacco, Mohammad Reza Saeb, Brijesh Sathian, Davide Sattin, Nikolaos Scarmeas, Falk Schwendicke, Rahman Shiri, Velizar Shivarov, Kibrom T. Sibhatu, Biagio Simonetti, Søren T. Skou, Joan B. Soriano, Ireneous N. Soyiri, Nicholas Steel, Simona Cătălina Stefan, Fridolin Steinbeis, Paschalis Steiropoulos, Leo Stockfelt, Saverio Stranges, Johan Sundström, Rafael Tabarés-Seisdedos, Arulmani Thiyagarajan, Roman Topor-Madry, Marcos Roberto Tovani-Palone, Nikolaos Tsilimparis, Brigid Unim, Marco Vacante, Jef Van den Eynde, Tommi Juhani Vasankari, Massimiliano Veroux, Jorge Hugo Villafañe, Francesco S. Violante, Yanzhong Wang, Ronny Westerman, Charles D. A. Wolfe, Grant M. A. Wyper, Sanni Yaya, Vesna Zadnik, Jean-David Zeitoun, Alimuddin Zumla, Alberto Freitas, Brecht Devleesschauwer

**Affiliations:** 1 MEDCIDS, Department of Community Medicine, Information and Health Decision Sciences, Faculty of Medicine, University of Porto, Porto, Portugal; 2 CINTESIS@RISE, Faculty of Medicine of the University of Porto, 4200-450 Porto, Portugal; 3 Public Health Unit, ULS Santo António, Porto, Portugal; 4 National School of Public Health. Instituto de Salud Carlos III, Madrid, Spain; 5 Scientific-Tools.Org, Bergamo, Italy; 6 Department of Public Health, Babeş-Bolyai University, Cluj-Napoca-Napoca, Romania; 7 RoNeuro Institute for Neurological Research and Diagnostic, Cluj-Napoca-Napoca, Romania; 8 Department for Exposure Assessment and Environmental Health Indicators, Germany Environment Agency, Berlin, Germany; 9 Clinic of Social and Family Medicine, School of Medicine, University of Crete, Crete, Greece; 10 Department of Healthcare, Faculty of Public Health, University of Vlora, Vlora, Albania; 11 Independent Researcher, Rome, Italy; 12 Institute of Gerontological Health Services and Nursing Research, Ravensburg-Weingarten University of Applied Sciences, Weingarten, Germany; 13 Centre for Disease Burden, Norwegian Institute of Public Health, Oslo, Norway; 14 Department of Epidemiology and Population Health, Faculty of Health Sciences, American University of Beirut, Beirut, Lebanon; 15 National Center for Epidemiology, Instituto de Salud Carlos III, Madrid, Spain; 16 Department of Public Health, Erasmus MC University Medical Center, Rotterdam, The Netherlands; 17 Department of Health Economics and Social Security, Faculty of Health Sciences, Jagiellonian University Medical College, Krakow, Poland; 18 Institute for Maternal and Child Health IRCCS Burlo Garofolo, Trieste, Italy; 19 Research group for Childhood Cancer, Danish Cancer Institute, Danish Cancer Society, Copenhagen, Denmark; 20 Medicine School, University of Applied and Environmental Sciences, Bogota, Colombia; 21 Department of International Health, Maastricht University, Care and Public Health Research Institute – CAPHRI, Maastricht, The Netherlands; 22 Department of Non-Communicable Diseases and Injuries, Santé Publique France, Saint-Maurice, France; 23 Department of Medicine, University College Cork, College Road, Cork City, Ireland; 24 Department of Geriatric Medicine, Mercy University Hospital, Grenville Place, Cork City, Ireland; 25 Department of Anatomy, Faculty of Medicine and Surgery, University of Malta, Msida, Malta; 26 Department of Biomedical and Dental Sciences and Morphofunctional Imaging, University of Messina, Messina, Italy; 27 School of Public Health, University College Cork, Cork, Ireland; 28 Department of Epidemiology and Public Health, Sciensano, Brussels, Belgium; 29 Department of Translational Physiology, Infectiology and Public Health, Ghent, Belgium

**Keywords:** European Union, Health status, Population health, Global Burden of Diseases, European Burden of Disease Network

## Abstract

**Background:**

The European Union (EU) faces many health-related challenges. Burden of diseases information and the resulting trends over time are essential for health planning. This paper reports estimates of disease burden in the EU and individual 27 EU countries in 2019, and compares them with those in 2010.

**Methods:**

We used the Global Burden of Disease 2019 study estimates and 95% uncertainty intervals for the whole EU and each country to evaluate age-standardised death, years of life lost (YLLs), years lived with disability (YLDs) and disability-adjusted life years (DALYs) rates for Level 2 causes, as well as life expectancy and healthy life expectancy (HALE).

**Results:**

In 2019, the age-standardised death and DALY rates in the EU were 465.8 deaths and 20,251.0 DALYs per 100,000 inhabitants, respectively. Between 2010 and 2019, there were significant decreases in age-standardised death and YLL rates across EU countries. However, YLD rates remained mainly unchanged. The largest decreases in age-standardised DALY rates were observed for “HIV/AIDS and sexually transmitted diseases” and “transport injuries” (each -19%). “Diabetes and kidney diseases” showed a significant increase for age-standardised DALY rates across the EU (3.5%). In addition, “mental disorders” showed an increasing age-standardised YLL rate (14.5%).

**Conclusions:**

There was a clear trend towards improvement in the overall health status of the EU but with differences between countries. EU health policymakers need to address the burden of diseases, paying specific attention to causes such as mental disorders. There are many opportunities for mutual learning among otherwise similar countries with different patterns of disease.

**Supplementary Information:**

The online version contains supplementary material available at 10.1186/s12889-024-18529-3.

## Key-points

• This article, systematically analysing GBD 2019 study estimates, presents an overview of the state of health in the European Union in 2019, compared to 2010.

• There was an improvement in the overall health status of the EU, despite substantial differences between Member States.

• Cardiovascular diseases and neoplasms are the major contributors to the overall burden of diseases in the EU in 2019.

• The age-standardised rate of years lived with disability due to mental disorders has been increasing and is expected to increase even more because of the COVID-19 pandemic.

• This report provides a framework upon which to base further region- and country-specific health policies and interventions, to support health planning and priority setting.

**Supplementary Information:**

The online version contains supplementary material available at 10.1186/s12889-024-18529-3.

## Introduction

The European Union (EU) faces many challenges that impact current and future population health, including complex issues such as population ageing, digital and green transitions, socio-economic challenges and the organisation of health systems. In addition, there are still significant differences in health status between EU countries which are associated with factors such as structural and budgetary differences, variations in the effectiveness of public health policies and health related risk factors [1–6]. In fact, health systems differ across the EU and, for instance, while the 2008 global financial crisis reduced annual health budgets, this did not happen uniformly. In addition, as population ageing advances, multimorbidity and frailty are becoming more common and need to be addressed to improve the well-being of EU countries [7–9].

According to Eurostat, life expectancy at birth in the EU was 81.0 years in 2019, with women living, on average, 5.5 years longer than men [4, 10]. Beyond life expectancy, population health can be summarised through combined health metrics such as health-adjusted life expectancy (HALE) and disability-adjusted life years (DALYs). DALYs consist of two components: (i) years of life lost (YLLs), which captures health loss due to premature mortality, and (ii) years lived with disability (YLDs), which quantifies health loss due to morbidity. A previous study showed a decline in YLD and DALY rates, an increase in life expectancy of 5.9 years and an increase in HALE of 4.6 years, on average, from 1990 to 2017 among EU-28 countries [11]. However, another study concluded that, despite the improvement in the health status of the EU, several central and eastern European countries had not experienced such pronounced gains in overall health in comparison to the EU-15 [12].

Accurate and timely data on mortality and morbidity, caused by diseases and injuries and their trends over time are essential to assess the impact of health strategies and assist policy makers in improving health planning and priority setting. This information can also be used to understand between-region variations, providing opportunities for mutual learning among EU countries. The Global Burden of Disease (GBD) study generates estimates of population health using a wide range of metrics, capturing the impact of diseases, injuries and risk factors on health. Furthermore, it allows for comprehensive comparisons over time and across countries. Burden of disease estimates are increasingly used in the EU and globally, as they provide a comprehensive and comparable picture of the overall population health status. An earlier analysis of the results from the GBD 2017 study for the EU countries examined changes since 2007 for the burden of diseases and injuries in the EU-28 in 2017 [13]. Between the release of the GBD 2017 and the GBD 2019 datasets, several improvements were made, including key demographic modelling steps, preferred/reference case definitions or measurement methods and the development of a Bayesian meta-regression tool, as well as the inclusion of more data sources and 12 new causes [14, 15].

In this paper, we analyse the GBD 2019 study estimates (focusing on deaths, YLDs, YLLs, DALYs, life expectancy and HALE) and compare the years 2019 and 2010 to describe the current health status of the EU. The aim of this study is to provide a picture of the state of health in the EU-27 countries in 2019, to examine how these have changed since 2010 and to highlight meaningful opportunities that exist to improve health across the continent.

## Methods

### Data source and overview of the GBD 2019 study

We obtained estimates from the 2019 GBD study for the EU-27 region and for the 27 EU countries individually. Considering the period of analysis, the 27 EU member states countries included were: Austria, Belgium, Bulgaria, Croatia, Cyprus, Czech Republic, Denmark, Estonia, Finland, France, Germany, Greece, Hungary, Ireland, Italy, Latvia, Lithuania, Luxembourg, Malta, Netherlands, Poland, Portugal, Romania, Slovakia, Slovenia, Spain and Sweden.

A detailed description of methods and results used in GBD 2019 has been published elsewhere [14–17]. In brief, the GBD 2019 study is a collaborative effort of more than 5,000 researchers, aiming to measure population health at global, regional and national levels by quantifying the burden of 369 diseases and injuries (i.e. 286 causes of death and 364 non-fatal causes) and 87 risk factors between 1990 and 2019 for 204 countries and territories. Several improvements were made in the GBD 2019 study, including key demographic modelling steps, reference case definitions or measurement methods and the Bayesian meta-regression tool. In addition, more data sources and 12 new causes were added to the GBD modelling framework, including pulmonary arterial hypertension, nine new sites of cancer, and two new sites of osteoarthritis (hand and other joints). The GBD produces estimates of incidence, prevalence, mortality, YLDs, YLLs, DALYs, life expectancy and HALE for the entire time span between 1990 and 2019. Cause-specific death rates and cause fractions are calculated using the Cause of Death Ensemble model (CODEm) and spatiotemporal Gaussian process regression. They are adjusted to match the total all-cause deaths calculated as part of the GBD population, fertility, and mortality estimates [15, 18].

DALYs consist of two main components: YLLs and YLDs. YLLs are calculated by multiplying the number of deaths of each age the remaining life expectancy (RLE) at age of death derived from the GBD standard life Table [19]. YLDs are estimated by multiplying the prevalence counts by the disability weight for each specific health outcome associated with a given disease or injury, with further adjustment for co-morbidity and severity. A Bayesian meta-regression modelling tool, DisMod-MR (Disease Modelling-Meta Regression) 2.1, ensures consistency between all epidemiologic metrics for most causes [16]. HALE accounts for years of life spent in good health and serves as a summary for both mortality and morbidity [13]. It thus corresponds to specific LE by age and geography, adjusted for the years spent living with disability and disability weights. All estimates are reported with their 95% uncertainty intervals (UI). UIs are propagated throughout the estimating process where 1000 draws are generated for each point estimate, and the 95% UIs are obtained by selecting the 2.5th and 97.5th percentiles of the draws. This approach ensures robustness in identifying meaningful differences and trends in health outcomes over time.

### Analytic strategies

The statistical significance of the difference between two estimates was defined as the absence of overlap between the 95% UI of those estimates. We analysed the overall (all ages and both sexes) and age group-specific rates for men and women. To analyse trends between 2010 and 2019, we relied on age-standardised rates and their relative changes since 2010. Difference between 2010 and 2019 was expressed in percentage change since 2010 (i.e. ${\% change}_{2010-2019}=\frac{{estimate}_{2019}-{estimate}_{2010}}{{estimate}_{2010}}\times 100$). In the GBD 2019 study, the same methodology is applied across years, including for 2010 and 2019. Age-standardisation is based on the GBD 2019 world standard population, which adjusts for differences in age distributions across populations, ensuring comparability between groups with different age structures. These rates were calculated using methodologies outlined in the GBD study, which provides upper and lower bounds of the estimates, allowing for a comprehensive analysis of trends over time.

The GBD arranges diseases and injuries (causes) into hierarchically nested categories in four levels of aggregation. At every level of aggregation, causes are mutually exclusive and collectively exhaustive. We extended this analysis focusing on each of the 22 Level 2 causes, and including seven Level 2 causes from Level 1 in the “communicable, maternal, neonatal and nutritional diseases” group (enteric infections, respiratory infections and tuberculosis, HIV/AIDs and sexually transmitted infections, maternal and neonatal disorders, neglected tropical diseases and malaria, nutritional deficiencies, other infectious diseases), 12 in the “non-communicable diseases” (NCDs) group (cardiovascular diseases, chronic respiratory diseases, diabetes and kidney diseases, digestive diseases, mental disorders, musculoskeletal disorders, neoplasm, neurological disorders, sense organ diseases, skin and subcutaneous diseases, substance use disorders, other NCDs), and three in the “injuries” group (self-harm and interpersonal, unintentional injuries and transport injuries). We considered Level 2 causes to focus the analysis on broad disease categories due to their policy implications. These causes represent broad disease categories where policy implications can result in benefits for all conditions summarized in these broader categories. We thus provide insights into the major drivers of health outcomes within the population.

All results are based on the estimates extracted from the Global Burden of Disease Results database and GBD Compare [20, 21]. All analyses were carried out with the open-source R Statistical Software (version 3.4, Foundation for Statistical Computing, Vienna, Austria) [22]. The GBD study adheres to the Guidelines for Accurate and Transparent Health Estimates Reporting (GATHER) standards developed by WHO and others [23].

## Results

### Overall disease burden in the European Union

In 2019, the EU-27 had a total of 5,354,279 (95% UI: 5,206,626; 5,502,691) all-cause deaths, yielding a crude death rate of 1040.3 (95% UI: 1011.6; 1069.1) per 100 000 inhabitants. The age-standardised death rate for all causes was 465.8 (95% UI: 451.2; 480.9) per 100,000 inhabitants in the EU, with high variability across countries, ranging from 894.8 per 100,000 in Bulgaria to 385.9 per 100,000 in Spain. The total number of all-cause DALYs was 157,884,271 (95% UI: 139,041,970; 178,511,173), with a crude rate of 30,675 (95% UI: 27,014; 34,683) per 100,000 inhabitants. The all-cause age-standardised DALY rate per 100 000 inhabitants in 2019 was 20 251.0 (95% UI: 17 408.1; 23 513.9).

**Fig. 1:**
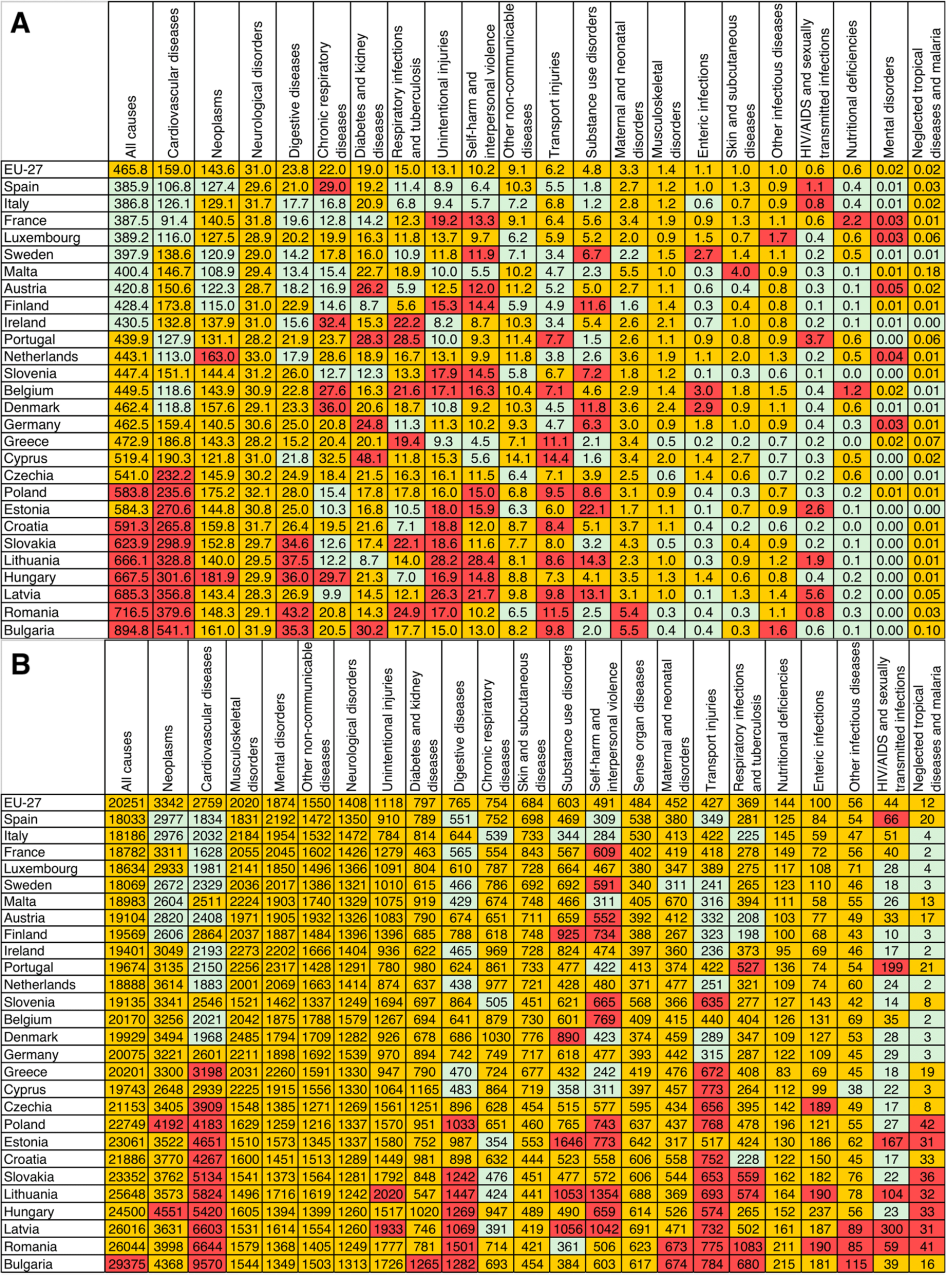
Age-standardised death (**A**) and DALY (**B**) rates (per 100 000 inhabitants) by the Level 2 causes for the European Union and for each country in 2019. *Footnote (to be included next to the figure)—Cells in green (or lighter grey) have a rate statistically significantly lower than EU, red (or darker) higher and yellow (or medium grey) without statistically significant differences*

Eight countries (Bulgaria, Romania, Latvia, Hungary, Lithuania, Slovakia, Croatia, and Poland) reported significantly higher (i.e., the lower limit of the individual country 95% UI was higher than the upper limit of the EU 95% UI) all-cause age-standardised death rates than the EU. In contrast, nine countries (Spain, Italy, France, Luxembourg, Sweden, Malta, Austria, Finland, and Ireland) had significantly lower rates than the EU (Fig. 1A; Table 1). The all-cause age-standardised death rate in the EU declined on average by 8.8% (95% UI: -11.7; -5.9), ranging from -4.8% (95% UI: -7.2; -2.3) in Greece to -18.5% (95% UI; -30.7%; -4.5%) in Lithuania.

**Table 1:** All-cause age-standardised death, YLL, YLD and DALY rates (per 100 000 inhabitants), life expectancy and healthy life expectancy for the European Union and for each EU country in 2019 and their percentage change between 2010 and 2019

	AS Death rate		AS YLL rate		AS YLD rate		AS DALY rate		Life expectancy		Health-adjusted life expectancy	
	2019	2010–2019 (%)	2019	2010–2019 (%)	2019	2010–2019 (%)	2019	2010–2019 (%)	2019	2010–2019 (%)	2019	2010–2019 (%)
**European Union**	**465.8 (451.2; 480.9)**	**-8.8 (-11.7; -5.9)**	**9564 (9158; 9997)**	**-12 (-15.7; -8.1)**	**10,687 (7908; 13,858)**	**0.6 (-0.1; 1.2)**	**20,251 (17,408; 23,514)**	**-5.8 (-8; -3.7)**	**81.0 (80.6; 81.3)**	**1.4 (1.0; 1.9)**	**69.8 (66.6; 72.7)**	**1.2 (0.7; 1.6)**
Austria	420.8 (413.4; 428.8)	-11.2 (-12.9; -9.4)	8360 (8154; 8584)	-14.9 (-17.2; -12.5)	10,744 (7927; 13,927)	-0.4 (-2; 1.2)	19,104 (16,292; 22,253)	-7.3 (-9.2; -5.6)	82.2 (82; 82.3)	1.7 (1.4; 2)	70.6 (67.2; 73.6)	1.6 (1.2; 1.9)
Belgium	449.5 (439.6; 460.2)	-9.4 (-11.6; -7.2)	9129 (8837; 9453)	-12.5 (-15.4; -9.4)	11,041 (8083; 14,321)	-2.1 (-3.7; -0.3)	20,170 (17,230; 23,435)	-7.1 (-9; -5.1)	81.4 (81.2; 81.6)	1.5 (1.2; 1.8)	69.7 (66.3; 72.8)	1.5 (1.1; 2)
Bulgaria	894.8 (744.3; 1070.7)	-7.1 (-23.7; 11)	19,339 (15,632; 23,799)	-9.4 (-27.9; 11.4)	10,036 (7413; 12,976)	0.3 (-1.5; 2.3)	29,375 (24,710; 34,547)	-6.3 (-18.7; 8)	73.3 (70.9; 75.7)	1.4 (-2; 4.9)	64.6 (61.4; 67.6)	1.3 (-1.9; 4.3)
Croatia	591.3 (486.8; 714.9)	-14.2 (-29.4; 3.6)	11,612 (9294; 14,433)	-16.7 (-33.4; 3.5)	10,274 (7607; 13,342)	0.2 (-2.1; 2.5)	21,886 (18,219; 26,054)	-9.5 (-19.5; 2)	78.7 (76.5; 80.8)	2.4 (-0.4; 5.2)	68.2 (64.8; 71.5)	2 (-0.5; 4.2)
Cyprus	519.4 (480.7; 563.4)	-14.4 (-21; -7.1)	9235 (8394; 10,194)	-12.4 (-20.6; -3)	10,508 (7709; 13,612)	0.4 (-1.1; 1.7)	19,743 (16,838; 22,880)	-6 (-10.7; -1.4)	80.8 (80; 81.6)	1.6 (0.6; 2.6)	69.9 (66.7; 72.9)	1.4 (0.5; 2.3)
Czechia	541 (457.8; 638.7)	-12.1 (-25.5; 3.6)	10,745 (8899; 12,953)	-15.2 (-29.7; 2.2)	10,409 (7680; 13,558)	0.2 (-1.8; 2.3)	21,153 (17,813; 24,971)	-8.2 (-16.7; 1.5)	79.5 (77.6; 81.3)	2.1 (-0.4; 4.4)	68.6 (65.1; 71.7)	1.7 (-0.4; 3.6)
Denmark	462.4 (449.3; 476.6)	-13.2 (-15.9; -10.5)	9162 (8798; 9566)	-14.9 (-18.4; -11.1)	10,768 (7956; 13,905)	-0.1 (-1.6; 1.6)	19,929 (17,111; 23,155)	-7.5 (-10; -5.2)	81.1 (80.8; 81.4)	2 (1.6; 2.4)	69.9 (66.7; 72.8)	1.7 (1.3; 2.2)
Estonia	584.3 (477.6; 707.2)	-12.8 (-28.8; 6)	13,026 (10,488; 16,078)	-15.4 (-32; 4.1)	10,035 (7400; 13,058)	-0.3 (-2.5; 1.8)	23,061 (19,316; 27,134)	-9.5 (-19.9; 2.3)	78 (75.6; 80.5)	2.4 (-0.9; 5.6)	68.1 (64.7; 71.4)	2.2 (-0.7; 5)
Finland	428.4 (414.9; 443.1)	-11.6 (-14.5; -8.5)	8765 (8419; 9144)	-15.4 (-18.9; -11.7)	10,805 (7991; 14,005)	-1.1 (-2.7; 0.6)	19,569 (16,724; 22,872)	-8.1 (-10.5; -5.9)	81.9 (81.5; 82.2)	1.8 (1.3; 2.3)	70.3 (67; 73.3)	1.8 (1.3; 2.3)
France	387.5 (380.3; 395.2)	-10.7 (-12.5; -8.9)	8282 (8061; 8526)	-13 (-15.5; -10.4)	10,499 (7719; 13,653)	0 (-1.8; 2)	18,782 (16,017; 21,919)	-6.2 (-8.2; -4.2)	82.9 (82.7; 83.1)	1.6 (1.3; 1.8)	71.5 (68.1; 74.5)	1.3 (0.9; 1.7)
Germany	462.5 (455.3; 471.1)	-5.2 (-6.7; -3.4)	9126 (8946; 9330)	-8.2 (-10.1; -6.2)	10,949 (8072; 14,255)	0.7 (-1.8; 3.2)	20,075 (17,158; 23,315)	-3.6 (-5.3; -1.7)	81.2 (81; 81.4)	0.9 (0.6; 1.1)	69.7 (66.4; 72.7)	0.6 (0.1; 1)
Greece	472.9 (461.8; 485.2)	-4.8 (-7.2; -2.3)	9543 (9206; 9929)	-6.1 (-9.6; -2.3)	10,658 (7853; 13,819)	-0.5 (-2.2; 1.3)	20,201 (17,423; 23,370)	-3.2 (-5.4; -1)	80.9 (80.7; 81.2)	0.7 (0.3; 1)	69.9 (66.7; 72.7)	0.7 (0.2; 1.1)
Hungary	667.5 (566.5; 785.6)	-13.1 (-26.1; 2.2)	14,296 (11,873; 17,181)	-15.9 (-30.1; 1)	10,204 (7532; 13,215)	0.4 (-1.6; 2.4)	24,500 (20,800; 28,629)	-9.8 (-18.7; 0.8)	76.6 (74.6; 78.6)	2.5 (-0.3; 5.1)	66.8 (63.6; 69.9)	2.1 (-0.2; 4.2)
Ireland	430.5 (416.4; 446)	-9.7 (-12.9; -6.4)	8320 (7935; 8752)	-14.5 (-18.6; -10.1)	11,081 (8177; 14,373)	0.2 (-1.5; 2)	19,401 (16,512; 22,745)	-6.7 (-9.4; -4.2)	82 (81.7; 82.4)	1.5 (1.1; 2)	70.4 (67; 73.3)	1.3 (0.7; 1.8)
Italy	386.8 (383.4; 390.1)	-9.2 (-10.1; -8.4)	7439 (7344; 7527)	-11.5 (-12.7; -10.4)	10,746 (7879; 14,084)	0.1 (-0.6; 0.8)	18,186 (15,294; 21,486)	-5 (-6.1; -4)	83.1 (83; 83.2)	1.2 (1.1; 1.4)	71.2 (67.8; 74.3)	1 (0.9; 1.2)
Latvia	685.3 (596.3; 797.8)	-15.7 (-26.6; -1.9)	15,938 (13,622; 18,804)	-19.9 (-31.5; -5.8)	10,078 (7427; 13,078)	-1 (-2.8; 0.9)	26,016 (22,399; 30,171)	-13.5 (-21.6; -3.9)	75.9 (73.8; 77.7)	3.5 (0.8; 6.1)	66.3 (63.2; 69.2)	3.3 (0.9; 5.6)
Lithuania	666.1 (565.5; 780)	-18.5 (-30.7; -4.5)	15,538 (13,039; 18,462)	-22.8 (-35; -8.1)	10,111 (7490; 13,073)	-2.1 (-4; -0.1)	25,648 (21,935; 29,654)	-15.8 (-24; -5.9)	76.2 (74.1; 78.3)	4.1 (1.2; 7)	66.6 (63.3; 69.6)	4 (1.4; 6.4)
Luxembourg	389.2 (353.4; 432.3)	-16.8 (-24.7; -7.4)	7794 (6939; 8847)	-16 (-25.7; -4.5)	10,840 (8014; 14,065)	-0.1 (-2; 1.8)	18,634 (15,642; 22,023)	-7.4 (-12.3; -1.9)	82.9 (81.8; 83.9)	2.3 (0.9; 3.7)	71 (67.5; 74.1)	1.9 (0.6; 3.1)
Malta	400.4 (365.8; 438.7)	-13.1 (-20.3; -5.3)	8350 (7400; 9502)	-12.7 (-22.6; -1.1)	10,633 (7845; 13,777)	-0.2 (-1.7; 1.3)	18,983 (16,094; 22,345)	-6.1 (-11.2; -0.6)	82.6 (81.6; 83.5)	1.8 (0.7; 3)	71.1 (67.7; 74.1)	1.5 (0.5; 2.6)
Netherlands	443.1 (432.3; 454.7)	-6.6 (-8.9; -4.1)	8503 (8200; 8844)	-9 (-12.3; -5.4)	10,385 (7710; 13,439)	2.3 (0.4; 4.5)	18,888 (16,190; 22,000)	-3.1 (-5.4; -0.8)	81.7 (81.5; 82)	2.1 (-0.2; 4.4)	70.6 (67.5; 73.4)	0.4 (-0.1; 0.9)
Poland	583.8 (504.2; 672.3)	-11.2 (-23.5; 2.1)	12,787 (10,894; 14,824)	-14.5 (-27.1; -0.5)	9963 (7364; 12,907)	0.1 (-0.7; 0.8)	22,749 (19,421; 26,563)	-8.7 (-16.6; -0.2)	78.1 (76.3; 79.9)	1.8 (1.5; 2.2)	68.1 (64.8; 71.2)	1.9 (-0.1; 3.8)
Portugal	439.9 (429.4; 451.3)	-12 (-14.3; -9.6)	8774 (8463; 9122)	-15.3 (-18.4; -11.8)	10,900 (8009; 14,120)	-2.2 (-3.8; -0.8)	19,674 (16,845; 22,930)	-8.5 (-10.7; -6.5)	81.7 (81.5; 82)	1.8 (1.5; 2.2)	70.2 (66.8; 73.2)	2 (1.6; 2.4)
Romania	716.5 (611.7; 835.4)	-12.2 (-25.1; 2.2)	16,199 (13,757; 19,117)	-14.6 (-27.5; 0.5)	9844 (7318; 12,819)	0.1 (-2; 2.2)	26,044 (22,457; 29,967)	-9.6 (-18.4; 0.7)	75.5 (73.5; 77.5)	2.4 (-0.3; 5)	66.4 (63.3; 69.2)	2.1 (-0.3; 4.4)
Slovakia	623.9 (511.6; 756.9)	-12.6 (-28.6; 6)	13,208 (10,559; 16,428)	-15 (-32.1; 5.3)	10,144 (7480; 13,113)	0 (-2.1; 2.1)	23,352 (19,472; 27,684)	-9.1 (-19.4; 3.3)	77.6 (75.2; 79.9)	2.3 (-0.8; 5.4)	67.6 (64.1; 70.8)	2 (-0.7; 4.5)
Slovenia	447.4 (362.1; 560.3)	-14.2 (-30.3; 6.9)	9023 (7218; 11,474)	-16.2 (-32.7; 6.4)	10,112 (7485; 13,119)	-2.5 (-4.4; -0.8)	19,135 (15,775; 23,040)	-9.5 (-18.5; 2.1)	81.4 (78.9; 83.7)	2.1 (-1; 5)	70.4 (66.7; 73.9)	2.2 (-0.6; 4.5)
Spain	385.9 (378.7; 393.6)	-8.9 (-10.6; -7)	7570 (7372; 7792)	-11.4 (-13.8; -8.8)	10,463 (7734; 13,596)	1.3 (-0.5; 3.1)	18,033 (15,282; 21,170)	-4.5 (-6.3; -2.6)	83.1 (82.9; 83.3)	1.2 (1; 1.5)	71.6 (68.3; 74.5)	0.9 (0.5; 1.3)
Sweden	397.9 (393.1; 403)	-8.5 (-9.9; -7.2)	7595 (7477; 7723)	-10.2 (-11.8; -8.5)	10,474 (7727; 13,605)	0.9 (-0.5; 2.2)	18,069 (15,379; 21,133)	-4.1 (-5.5; -2.8)	82.8 (82.7; 83)	1.2 (1; 1.4)	71.4 (68.1; 74.3)	0.9 (0.6; 1.2)

In 2019, compared to the EU, all-cause age-standardised DALY rates were significantly higher only in Bulgaria (Fig. 1B; Table 1). All-cause age-standardised DALY rates have declined significantly since 2010 (i.e., the upper limit of the 95% UI below zero) in most countries, except for Bulgaria, Croatia, Czechia, Estonia, Hungary, Romania, Slovakia, and Slovenia. Whilst most countries showed a decreasing trend in all-cause age-standardised YLL rates, no significant changes were found in all-cause age-standardised YLD rates between 2010 and 2019. Only Belgium, Lithuania, Portugal, and Slovenia experienced significant declines, while the Netherlands experienced significant increases in YLD rates (Table 1).

In 2019, life expectancy in the EU at birth was 81.0 years, ranging from 73.3 years in Bulgaria to 83.1 years in Italy and Spain. All countries experienced improvements in life expectancy between 2010 and 2019, with Lithuania having the highest increase (4.5%) and the EU-27 showing a 1.4% increase (from 79.8 years to 81.0 years). HALE at birth for the EU in 2019 was 69.8 years, ranging from 64.6 years in Bulgaria to 71.6 years in Spain, with HALE at birth improving by 1.2% between 2010 (i.e. 69.0 years) and 2019 across the EU-27. However, the gap between life expectancy and HALE widened from 10.8 years (13.6% of LE) in 2010 to 11.2 in 2019 (13.8% of LE), which suggests that YLDs represent a growing share of DALY rates.

### Overall disease burden by age and sex

**Fig. 2:**
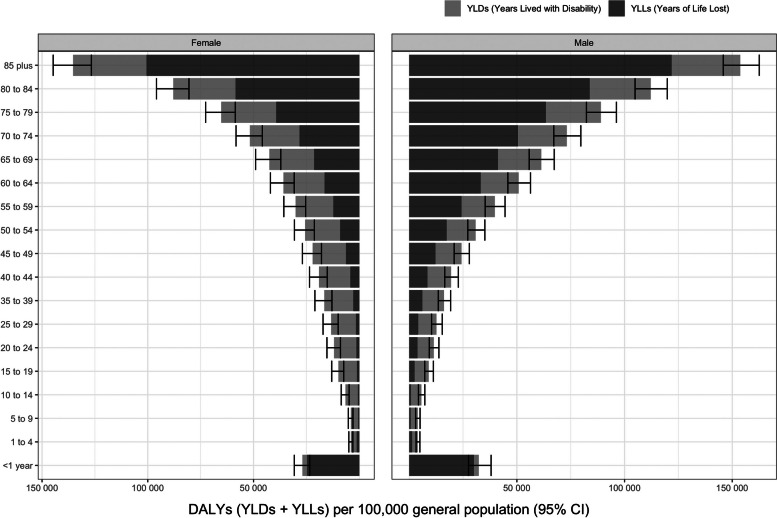
Disability adjusted life years (DALYs) per 100,000 inhabitants, split into Years of life lost (YLL) and Years lived with disability (YLD), by sex and age group in the European Union in 2019. The error bars indicate the 95% uncertainty interval around the DALYs estimates

DALY rates increased similarly with age in both males and females. However, across the EU in 2019, for most age groups, DALY rates were higher among males than females (Fig. 2). For males, DALY rates were mostly driven by YLLs in those aged above 44 years of age and by YLDs among younger (< 44 years old) age groups. For females, this cut-off occurred at a more advanced age, with DALY rates mostly driven by YLLs in groups aged above 64 years. YLLs dominated over YLDs in both sexes particularly in age extremes, i.e. younger and older age groups (Fig. 2).

### Main causes of ill health

In 2019, the age-standardised death rates for cardiovascular diseases were significantly higher than the EU rate (159.0; 95% UI 142.2; 169.2) in most Central and Eastern European countries, with the highest values in Bulgaria, Romania, and Latvia, and significantly lower than the EU rate in some Western European countries (Fig. 1A). A similar geographic pattern was observed for age-standardised DALY rates for cardiovascular diseases, the second leading cause of age-standardised DALY in the EU (Fig. 1B).

Compared to the EU, the age-standardized death (143.6; 95% UI 133.8; 150.1) and DALY (3,342; 95% UI 3,175; 3,505) rates for neoplasms in 2019 were significantly lower in Spain, Sweden, Malta, Austria, and Finland (Fig. 1A and B). Hungary and Netherlands showed a significantly higher age-standardised death rate, with Hungary and Poland having a significantly higher age-standardized DALY rate (Fig. 1A and B). In fact, the age-standardised death rate in Hungary was almost two times higher than in France. Neoplasms were the leading cause of age-standardized DALY and the second highest cause of age-standardised mortality across the EU in 2019.

Digestive diseases are another example of high variability in death rates in EU countries. The highest (in Romania) to the lowest (in Malta) age-standardised death rates ratio is over 3.2. Additionally, countries of Central and Eastern Europe (Romania, Lithuania, Bulgaria, Hungary, Slovakia, Latvia, Poland) had significantly higher DALY rates than the EU rate.

**Fig. 3:**
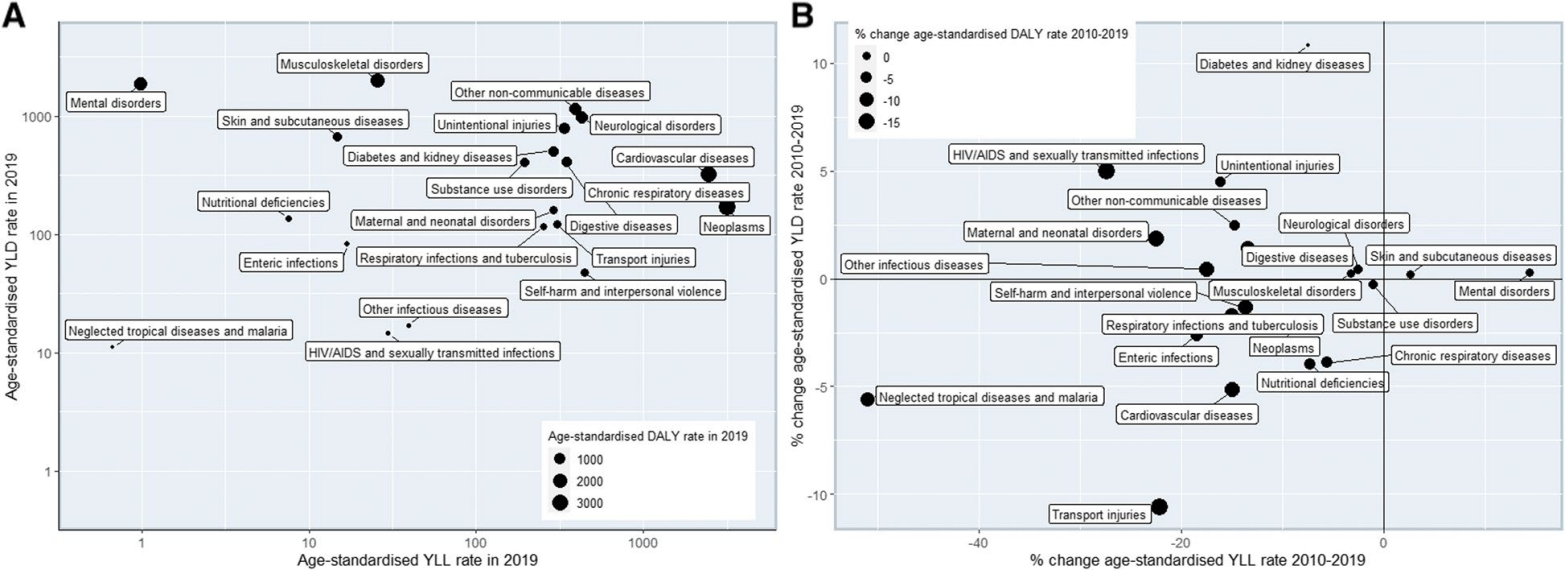
Age-standardised YLL, YLD and DALY rates in 2019 (A), as well as changes (in %) between 2010 and 2019 (B) for the Level 2 causes in the European Union

Figure 1A and B show the age-standardised death and DALY rates, respectively, for the EU and each EU country in 2019, for all Level 2 causes, comparing each country with the EU. Causes had different patterns across EU countries. For example, for HIV/AIDS and sexually transmitted infections, Latvia and Portugal had the highest age-standardised death and DALY rates, with more than five times the EU. The contribution of fatal and non-fatal components of age-standardised DALY rates varied substantially across Level 2 causes (Fig. 3A). For neoplasms and cardiovascular diseases, YLLs contributed more than YLDs, while for musculoskeletal and mental disorders, the total DALYs were almost exclusively YLDs.

Figure 3B highlights the relative change in age-standardised YLL, YLD and DALY rates between 2010 and 2019 for Level 2 causes. Age-standardised YLL rates declined for all causes except for mental disorders (14.5% increase) and skin and subcutaneous diseases (2.6% increase), while Level 2 causes were quite evenly split between increases and decreases for age-standardised YLD rates. The largest decreases in age-standardised DALY rates were observed for HIV/AIDS and sexually transmitted diseases (-19.2%) and transport injuries (-19.1%). On the other hand, only diabetes and kidney diseases showed a significant increase (3.5%) for age-standardised DALY rates in the EU between 2010 and 2019, mainly due to the age-standardised YLD rate increase. Finally, it is worth mentioning that mental disorders showed a non-significant increase for age-standardised DALY rates between 2010 and 2019 and this increase was mainly due to YLL rates, although there was also an increase in age-standardised YLD rates.

## Discussion

This study presents an overview of the state of health of the EU-27 and individual Member States in 2019, comparing the findings with data from 2010 to examine changes over the last decade. The results indicate that most countries experienced a significant, albeit varied, reduction in all-cause, age-standardised mortality and YLL rates over this period, although this pattern differed by country and region. During this time period, there were no substantial changes in all-cause age-standardised YLD rates, with the increase in the Netherlands being the most notable. The EU all-cause, age-standardised DALY rate fell by 5.8% over this period, mirroring global trends [15].

The observed variability in all-cause age-standardized death rates across countries in 2019 indicates that there are geographical clusters of mortality in the EU. The pattern of mortality burden clusters with a clear geographical variation across the EU was also observed for life expectancy rates and HALE measures. This pattern has been previously highlighted [13]. However, despite recent progress to reduce these differences, its persistence suggests that improvements may not continue uniformly across the EU without enhanced, combined and coordinated efforts to address a wide range of inequalities across health determinants, including socioeconomic factors.

Neoplasms and cardiovascular diseases were the leading causes for the burden of disease in the EU in 2019; both are attributable to the behavioural risk factors and depend on early diagnosis, treatment and management of risk factors. Inherently, these are among the costliest diseases for EU countries [24]. Additionally, with population ageing, NCDs are expected to increase over time and represent a greater proportion of overall deaths with higher mortality rates associated with cancer and cardiovascular disease relative to communicable diseases [25]. A recent analysis of changes in mortality and disability, comparing data from the GBD 1990–2019, confirmed this trend, finding that there has been an overall increase in disease burden among older Europeans during this time period, primarily driven by cardiovascular diseases [26]. These changes have not been homogeneous across the EU. As structures and systems take time to adapt to such changes, it suggests that existing differences may magnify if intervention strategies are not urgently introduced. Examining age-standardised DALY due to cancer, rates in the EU were between those of China (higher rate) and the United States of America (lower rate) [27]. EU countries had an estimated cancer burden of 4 million new cases annually in 2020, with cancer disproportionately affecting older Europeans and those living in Eastern EU Member States [28]. Future interventions must be designed to address the main drivers of NCDs, including population ageing, changes in population structure, and improvements in population-level risk factors, also considered in the Europe's Beating Cancer Plan. These must also address reasons for important differences across European regions. For example, despite the existence of cancer screening programmes across EU countries, differences in uptake of cancer screening varies according to socioeconomic factors; inequalities including lower household income, higher unemployment, and lower levels of educational attainment are associated with reduced uptake, especially in Eastern EU member states [29]. A similar trend is seen for cardiovascular diseases. EU member states with lower income levels and greater degrees of socioeconomic inequalities have disproportionately higher incidence rates and a greater burden of cardiovascular disease [30].

In this study, age-standardised death rates between countries varied widely by disease. For example, the results draw attention to the preventable high rates of self-harm and interpersonal violence across the EU in 2019. These mainly affected younger age groups. Although rates vary across the EU, we observed a geographical pattern with higher rates in the Baltic region. These differences in self-harm have been shown previously and likely relate to differences in the burden of mental disorders across the EU [31]. Baltic countries have historically had the highest rates of alcohol-related mortality and suicide, as well as a high burden of mental and behavioural disorders [32].

Infectious diseases, in general, represented a small share of age-standardised death rates until 2019, presenting an optimistic scenario regarding these most preventable diseases. There was, however, considerable heterogeneity in age-standardised death rates for some infectious diseases such as HIV and sexually transmitted infections, which despite overall low rates, had prominent outliers with relatively high rates in Latvia and Portugal. This highlights the importance of national preventive programmes that tackle the different transmission pathways, alongside with strengthening of surveillance systems [33].

Besides this heterogeneity, such infectious diseases showed an increased age-standardised YLL rate. This will be difficult to overcome without tailored health policies as the incidence of HIV is still increasing in several EU countries [34]. Moreover, infectious diseases are likely to represent a growing share of total disease burden following the COVID-19 pandemic, and will likely be of great importance in future GBD revisions.

Regarding DALYs, remarkable regional differences were found in cardiovascular diseases, self-harm and transport injuries, which were significantly higher in Eastern EU countries. Mental disorders were the fourth highest cause of age-standardised DALY rates and did not show a decrease over recent years. In fact, they showed a non-significant increase, mainly due to a remarkable increase in YLL. These conditions also represent one of the leading causes of YLD, which has been rising over recent years and has increased even more following the COVID-19 pandemic [35, 36]. Additionally, self-harm and interpersonal violence may also be linked to mental disorders, as an example of interacting causes. Thus, viewed as a whole, mental health disorders and other related possible outcomes such as self-harm and mortality linked to mental disorders, deserve special attention in line with WHO priorities [37].

The age-standardised YLD estimates generated by the GBD 2019 study show slight variation over time and across geographic areas and are subject to large levels of uncertainty. The former is mainly driven by the fact that the GBD severity distributions do not vary over time and space [38], essentially reducing differences in YLD rates to differences in the underlying prevalence estimates. Since prevalence data are typically sparser and more uncertain than mortality data, the modelled prevalence estimates further tend to smooth out temporal and spatial heterogeneity. In parallel, EU countries would need to improve the quality and performance of their health information systems, strengthening and integrating data available through disease registers, claims data, primary care data, hospital discharge data and health surveys.

### Strengths and limitations

This study is important and timely as it reflects the state of health in the EU prior to a number of major changes, including the COVID-19 pandemic and Brexit (the departure of the UK from the EU), and therefore will likely be important for policy-makers to understand the state of health of Europe at this pivotal moment in time. Although the UK is not included in the analysis and that potential adverse effects of Brexit on the health of the UK have been discussed [39], less is known about how it could impact the remaining EU-27. To date, there has been wide variation in the resilience and responses of health systems and governments to the pandemic across the EU, which replicates many of the regional variations presented in this study of the state of health of the EU. Comparing the results of this study with post-pandemic and post-Brexit GBD data will therefore be crucial to assess the impact of these ‘shocks’ on the health of EU citizens. Moreover, it could be pivotal for policy makers to address in future studies. Another strength of this study is that it provides estimates at the national level for EU countries for which burden of disease studies are lacking or are scarce and can support priority setting and resource allocations. This study used estimates provided by the GBD 2019 study and hence shares some limitations with other GBD studies, predominantly related mostly to the availability and quality of primary data, in particular for morbidity, which might not be homogeneous across EU countries. Moreover, there are some limitations pertaining to this paper related to: (1) the study design as it is a descriptive study, does not aim to estimate the effect of EU level policies; (2) timeline (as it provides an overall EU-level assessment across 10 years and excludes in-depth national assessment taking into account the year of accession to the EU); and (3) data availability. In addition, GBD metrics apply the same disability weights for all countries and regions. Such limitations have been widely discussed in the literature [15, 38].

Regarding the age-standardisation, it is also essential to highlight that while it is essential to ensure a global and comparable age standardisation, the used world standard population by GBD instead of a European standard population may change the ranking of causes [40].

## Conclusions

In conclusion, although population health in the EU has been improving, large differences between countries persist. Health outcomes remain much better in Western or Southern Europe (e.g. Spain, Italy or France) than in Central and Eastern Europe (e.g. Bulgaria or Romania) or the Baltic states (e.g. Latvia or Lithuania). NCDs, particularly neoplasms and cardiovascular diseases continue to be the leading causes of disease burden. This study suggests that addressing the prevalence and incidence of diseases and injuries should be a priority for EU health policy makers, emphasising reducing health inequalities across the block. Attention must be paid to specific causes, including mental disorders, given their impact on YLD [41]. This study highlights that there are many opportunities for mutual learning among otherwise similar EU countries with different patterns of disease and injury.

### Supplementary Information

Supplementary Material 1.

## Data Availability

The datasets analysed during the current study are publicly available in the GBD 2019 Results Tool and GBD 2019 Compare repositories (https://vizhub.healthdata.org/gbd-results/ and https://vizhub.healthdata.org/gbd-compare, respectively).
