# Supplementary material for: The state of health in the European Union (EU-27) in 2019: a systematic analysis for the Global Burden of Disease study 2019
Source: BMC Public Health. 2024 May 22;24:1374. doi: 10.1186/s12889-024-18529-3 (PMC11110444; doi:10.1186/s12889-024-18529-3)
Supplement: Supplementary file 1 — Supplementary Material 1. [file 12889_2024_18529_MOESM1_ESM.docx]

## Further Authors’ Contributions Appendix

### Providing data or critical feedback on data sources

Cristiana Abbafati, Hassan Abolhassani, Victor Adekanmbi, Sepideh Ahmadi, François Alla, Sofia Androudi, Seth Christopher Yaw Appiah, Olatunde Aremu, Sameh Attia, Marcel Ausloos, Maciej Banach, Till Winfried Bärnighausen, Sandra Barteit, Sanjay Basu, Derrick A Bennett, Boris Bikbov, Mahdi Bohluli, Israel Júnior Borges do Nascimento, Nicola Luigi Bragazzi, Daniela Calina, Vijay Kumar Chattu, Rajiv Chowdhury, Natália Cruz-Martins, Giovanni Damiani, Andreas K Demetriades, Nikolaos Dervenis, Mostafa Dianatinasab, Diana Dias da Silva, Abdel Douiri, David Edvardsson, Adeniyi Francis Fagbamigbe, Carla Sofia e Sá Farinha, Seyed-Mohammad Fereshtehnejad, Alberto Freitas, Peter Andras Gaal, Gus Gazzard, Nermin Ghith, Simona Giampaoli, James C Glasbey, Josep Maria Haro, Jan Hartvigsen, Mehdi Hosseinzadeh, Salman Hussain, Gaetano Isola, Jost B Jonas, Tamas Joo, Jacek Jerzy Jozwiak, Mikk Jürisson, Zubair Kabir, Moien AB Khan, Khaled Khatab, Om P Kurmi, Dian Kusuma, Anders O Larsson, Savita Lasrado, Mall Leinsalu, Stefan Lorkowski, Ronan A Lyons, Colm McAlinden, Enkeleint A Mechili, Ritesh G Menezes, Atte Meretoja, Bartosz Miazgowski, Shafiu Mohammed, Ali H Mokdad, Lorenzo Monasta, Joana Morgado-da-Costa, Francesk Mulita, Christopher J L Murray, Ionut Negoi, Ruxandra Irina Negoi, Che Henry Ngwa, Bogdan Oancea, Raffaele Palladino, Songhomitra Panda-Jonas, Shahina Pardhan, Jay Patel, José L Peñalvo, Renato B Pereira, Dietrich Plass, Maarten J Postma, Amir Masoud Rahmani, Chythra R Rao, Salman Rawaf, Reza Rawassizadeh, João Vasco Santos, Rodrigo Sarmiento-Suarez, Brijesh Sathian, Falk Schwendicke, Fridolin Steinbeis, Gerhard Sulo, Johan Sundström, Rafael Tabarés-Seisdedos, Roman Topor-Madry, Marcos Roberto Tovani-Palone, Nikolaos Tsilimparis, Jef Van den Eynde, Tommi Juhani Vasankari, Massimiliano Veroux, Ronny Westerman, Charles D A Wolfe, Grant M A Wyper, Sanni Yaya, and Vesna Zadnik.

### Developing methods or computational machinery

Mahdi Bohluli, Periklis Charalampous, Brecht Devleesschauwer, Mostafa Dianatinasab, Adeniyi Francis Fagbamigbe, Federica Gazzelloni, Nermin Ghith, Simon I Hay, Mehdi Hosseinzadeh, An Li, Ali H Mokdad, Christopher J L Murray, Amir Masoud Rahmani, Reza Rawassizadeh, João Vasco Santos, Roman Topor-Madry, Nikolaos Tsilimparis, Jorge Hugo Villafañe, Ronny Westerman, Grant M A Wyper,

### Providing critical feedback on methods or results

Cristiana Abbafati, Hassan Abolhassani, Victor Adekanmbi, Keivan Ahmadi, Adel Al-Jumaily, Robert Ancuceanu, Catalina Liliana Andrei, Tudorel Andrei, Josep M Antó, Seth Christopher Yaw Appiah, Olatunde Aremu, Ashokan Arumugam, Sameh Attia, Avinash Aujayeb, Marcel Ausloos, Maciej Banach, Till Winfried Bärnighausen, Sandra Barteit, Sanjay Basu, Bernhard T Baune, Derrick A Bennett, Mahdi Bohluli, Israel Júnior Borges do Nascimento, Nicola Luigi Bragazzi, Hermann Brenner, Danilo Buonsenso, Reinhard Busse, Daniela Calina, Giulio Castelpietra, Alberico L Catapano, Joht Singh Chandan, Periklis Charalampous, Vijay Kumar Chattu, Rajiv Chowdhury, Sheng-Chia Chung, Natália Cruz-Martins, Sarah Cuschieri, Giovanni Damiani, Andreas K Demetriades, Nikolaos Dervenis, Mostafa Dianatinasab, Diana Dias da Silva, Abdel Douiri, David Edvardsson, Francesco Esposito, Adeniyi Francis Fagbamigbe, Seyed-Mohammad Fereshtehnejad, João C Fernandes, Pietro Ferrara, Florian Fischer, Alberto Freitas, Peter Andras Gaal, Lucia Galluzzo, Mariana Gaspar Fonseca, Federica Gazzelloni, Nermin Ghith, Paramjit Singh Gill, James C Glasbey, Michal Grivna, Juanita A Haagsma, Abdul Hafiz, Romana Haneef, Jan Hartvigsen, Behzad Heibati, David Hillus, Mehdi Hosseinzadeh, Mihaela Hostiuc, Rok Hrzic, Salman Hussain, Gaetano Isola, Olatunji Johnson, Jost B Jonas, Tamas Joo, Jacek Jerzy Jozwiak, Mikk Jürisson, Zubair Kabir, Marina Karanikolos, Joonas H Kauppila, Moien AB Khan, Khaled Khatab, Katarzyna Kissimova-Skarbek, Miloslav Klugar, Ai Koyanagi, Om P Kurmi, Dian Kusuma, Carlo La Vecchia, Demetris Lamnisos, Heidi Jane Larson, Savita Lasrado, Paolo Lauriola, Jeffrey V Lazarus, Caterina Ledda, Miriam Levi, An Li, Giancarlo Logroscino, Stefan Lorkowski, Joana A Loureiro, Ronan A Lyons, Azeem Majeed, Alexander G Mathioudakis, Colm McAlinden, John J McGrath, Enkeleint A Mechili, Ritesh G Menezes, Alexios-Fotios A Mentis, Atte Meretoja, Tomislav Mestrovic, Junmei Miao Jonasson, Tomasz Miazgowski, Andreea Mirica, Shafiu Mohammed, Ali H Mokdad, Lorenzo Monasta, Ute Mons, Joana Morgado-da-Costa, Francesk Mulita, Christopher J L Murray, Ionut Negoi, Ruxandra Irina Negoi, Evangelia Nena, Che Henry Ngwa, Bogdan Oancea, Frank B Osei, Adrian Otoiu, Raffaele Palladino, Songhomitra Panda-Jonas, Shahina Pardhan, Jay Patel, Mihaela Paun, Paolo Pedersini, José L Peñalvo, Renato B Pereira, Jorge Pérez-Gómez, Ionela-Roxana Petcu, Marina Pinheiro, Dietrich Plass, Maarten J Postma, Amir Masoud Rahmani, Chythra R Rao, Salman Rawaf, Reza Rawassizadeh, Abanoub Riad, Mohammad Reza Saeb, João Vasco Santos, Rodrigo Sarmiento-Suarez, Brijesh Sathian, Nikolaos Scarmeas, Falk Schwendicke, Rahman Shiri, Velizar Shivarov, Kibrom T Sibhatu, Søren T Skou, Joan B Soriano, Ireneous N Soyiri, Simona Cătălina Ștefan, Fridolin Steinbeis, Saverio Stranges, Gerhard Sulo, Rafael Tabarés-Seisdedos, Arulmani Thiyagarajan, Marcos Roberto Tovani-Palone, Nikolaos Tsilimparis, Jef Van den Eynde, Massimiliano Veroux, Jorge Hugo Villafañe, Francesco S Violante, Yanzhong Wang, Ronny Westerman, Charles D A Wolfe, Grant M A Wyper, Sanni Yaya, and Vesna Zadnik.

### Drafting the work or revising is critically for important intellectual content

Cristiana Abbafati, Hassan Abolhassani, Victor Adekanmbi, Sepideh Ahmadi, Jordi Alonso, Robert Ancuceanu, Catalina Liliana Andrei, Tudorel Andrei, Josep M Antó, Seth Christopher Yaw Appiah, Olatunde Aremu, Benedetta Armocida, Johan Ärnlöv, Ashokan Arumugam, Sameh Attia, Avinash Aujayeb, Marcel Ausloos, Jose L Ayuso-Mateos, Maciej Banach, Till Winfried Bärnighausen, Francesco Barone-Adesi, Sandra Barteit, Sanjay Basu, Bernhard T Baune, Massimiliano Beghi, Luis Belo, Boris Bikbov, Antonio Biondi, Israel Júnior Borges do Nascimento, Nicola Luigi Bragazzi, Tasanee Braithwaite, Hermann Brenner, Danilo Buonsenso, Daniela Calina, Giulia Carreras, Márcia Carvalho, Giulio Castelpietra, Alberico L Catapano, Maria Sofia Cattaruzza, Joht Singh Chandan, Vijay Kumar Chattu, Simiao Chen, Rajiv Chowdhury, Hanne Christensen, Joao Conde, Barbara Corso, Natália Cruz-Martins, Sarah Cuschieri, Giovanni Damiani, Alejandro de la Torre-Luque, Andreas K Demetriades, Nikolaos Dervenis, Brecht Devleesschauwer, Mostafa Dianatinasab, Diana Dias da Silva, David Edvardsson, Luchuo Engelbert Bain, Francesco Esposito, Adeniyi Francis Fagbamigbe, Seyed-Mohammad Fereshtehnejad, João C Fernandes, Pietro Ferrara, Florian Fischer, Alberto Freitas, Peter Andras Gaal, Silvano Gallus, Lucia Galluzzo, Mariana Gaspar Fonseca, Federica Gazzelloni, Nermin Ghith, Alessandro Gialluisi, Paramjit Singh Gill, James C Glasbey, Giuseppe Gorini, Diana Alecsandra Grad, Michal Grivna, Juanita A Haagsma, Abdul Hafiz, Romana Haneef, Josep Maria Haro, Jan Hartvigsen, Simon I Hay, David Hillus, Sorin Hostiuc, Rok Hrzic, Salman Hussain, Gaetano Isola, Olatunji Johnson, Jost B Jonas, Tamas Joo, Jacek Jerzy Jozwiak, Mikk Jürisson, Marina Karanikolos, Joonas H Kauppila, Moien AB Khan, Khaled Khatab, Katarzyna Kissimova-Skarbek, Miloslav Klugar, Ai Koyanagi, Dian Kusuma, Carlo La Vecchia, Ben Lacey, Anders O Larsson, Savita Lasrado, Caterina Ledda, Paul H Lee, Mall Leinsalu, Matilde Leonardi, Miriam Levi, An Li, Christine Linehan, Stefan Lorkowski, Joana A Loureiro, Ronan A Lyons, Áurea M Madureira-Carvalho, Alexander G Mathioudakis, Colm McAlinden, John J McGrath, Enkeleint A Mechili, Ritesh G Menezes, Alexios-Fotios A Mentis, Atte Meretoja, Tuomo J Meretoja, Tomislav Mestrovic, Tomasz Miazgowski, Shafiu Mohammed, Ali H Mokdad, Lorenzo Monasta, Stefania Mondello, Ute Mons, Joana Morgado-da-Costa, Francesk Mulita, Christopher J L Murray, Ionut Negoi, Ruxandra Irina Negoi, Serban Mircea Negru, Che Henry Ngwa, Isabel Noguer, Nurulamin M Noor, George Ntaios, Bogdan Oancea, Rónán O'Caoimh, Frank B Osei, Adrian Otoiu, Alicia Padron-Monedero, Songhomitra Panda-Jonas, Shahina Pardhan, Jay Patel, Paolo Pedersini, José L Peñalvo, Umberto Pensato, Renato B Pereira, Jorge Pérez-Gómez, Norberto Perico, Ionela-Roxana Petcu, Dietrich Plass, Maarten J Postma, Alberto Raggi, Chythra R Rao, Salman Rawaf, Giuseppe Remuzzi, Abanoub Riad, Simona Sacco, João Vasco Santos, Rodrigo Sarmiento-Suarez, Davide Sattin, Nikolaos Scarmeas, Kibrom T Sibhatu, Biagio Simonetti, Søren T Skou, Joan B Soriano, Ireneous N Soyiri, Nicholas Steel, Simona Cătălina Ștefan, Fridolin Steinbeis, Paschalis Steiropoulos, Leo Stockfelt, Saverio Stranges, Gerhard Sulo, Johan Sundström, Arulmani Thiyagarajan, Roman Topor-Madry, Marcos Roberto Tovani-Palone, Nikolaos Tsilimparis, Brigid Unim, Marco Vacante, Jef Van den Eynde, Tommi Juhani Vasankari, Massimiliano Veroux, Francesco S Violante, Yanzhong Wang, Ronny Westerman, Grant M A Wyper, Sanni Yaya, Jean-David Zeitoun, and Alimuddin Zumla.

### Managing the estimation or publications process

Israel Júnior Borges do Nascimento, Simon I Hay, Ali H Mokdad, Christopher J L Murray, Che Henry Ngwa, and João Vasco Santos.
